# Folate Insufficiency Due to MTHFR Deficiency Is Bypassed by 5-Methyltetrahydrofolate

**DOI:** 10.3390/jcm9092836

**Published:** 2020-09-02

**Authors:** Maša Vidmar Golja, Alenka Šmid, Nataša Karas Kuželički, Jurij Trontelj, Ksenija Geršak, Irena Mlinarič-Raščan

**Affiliations:** 1 Research Unit, Department of Obstetrics and Gynecology, University Medical Centre Ljubljana, Šlajmerjeva 3, 1000 Ljubljana, Slovenia; masa.vidmar.golja@ffa.uni-lj.si (M.V.G.); ksenija.gersak@mf.uni-lj.si (K.G.); 2 Faculty of Pharmacy, University of Ljubljana, Aškerčeva 7, 1000 Ljubljana, Slovenia; alenka.smid@ffa.uni-lj.si (A.Š.); Natasa.KarasKuzelicki@ffa.uni-lj.si (N.K.K.); Jurij.Trontelj@ffa.uni-lj.si (J.T.); 3 Faculty of Medicine, University of Ljubljana, Vrazov trg 2, 1000 Ljubljana, Slovenia

**Keywords:** 5,10-methylenetetrahydrofolate reductase (MTHFR) polymorphisms, folate supplementation, folic acid (FA), 5-methyl-tetrahydrofolate (5-Me-THF)

## Abstract

Adequate levels of folates are essential for homeostasis of the organism, prevention of congenital malformations, and the salvage of predisposed disease states. They depend on genetic predisposition, and therefore, a pharmacogenetic approach to individualized supplementation or therapeutic intervention is necessary for an optimal outcome. The role of folates in vital cell processes was investigated by translational pharmacogenetics employing lymphoblastoid cell lines (LCLs). Depriving cells of folates led to reversible S-phase arrest. Since 5,10-methylenetetrahydrofolate reductase (MTHFR) is the key enzyme in the biosynthesis of an active folate form, we evaluated the relevance of polymorphisms in the MTHFR gene on intracellular levels of bioactive metabolite, the 5-methyltetrahydrofolate (5-Me-THF). LCLs (*n* = 35) were divided into low- and normal-MTHFR activity groups based on their genotype. They were cultured in the presence of folic acid (FA) or 5-Me-THF. Based on the cells’ metabolic activity and intracellular 5-Me-THF levels, we conclude supplementation of FA is sufficient to maintain adequate folate level in the normal MTHFR activity group, while low MTHFR activity cells require 5-Me-THF to overcome the metabolic defects caused by polymorphisms in their MTHFR genes. This finding was supported by the determination of intracellular levels of 5-Me-THF in cell lysates by LC-MS/MS. FA supplementation resulted in a 2.5-fold increase in 5-Me-THF in cells with normal MTHFR activity, but there was no increase after FA supplementation in low MTHFR activity cells. However, when LCLs were exposed to 5-Me-THF, a 10-fold increase in intracellular levels of this metabolite was determined. These findings indicate that patients undergoing folate supplementation to counteract anti-folate therapies, or patients with increased folate demand, would benefit from pharmacogenetics-based therapy choices.

## 1. Introduction

Micronutrient folate (vitamin B_9_) has a vital role in human health and disease and is essential for several crucial cellular processes, including DNA synthesis, amino acid interconversion, various methylation reactions, and consequently, for normal cell division and growth. Folate deficiency is associated with several congenital malformations, such as neural tube defects (NTDs), congenital heart defects, orofacial clefts, pregnancy-related complications, cardiovascular diseases, various psychiatric diseases, and cancer [1,2,3]. By contrast, excessive folate intake has also been associated with adverse health effects, such as increased risk for autism spectrum disorder and other negative neurocognitive development outcomes [4,5]. Hence, assuring adequate folate supply is essential for maintaining homeostasis of the organism and the absence of malformations or disease conditions. Three main factors influencing folate levels in individuals include dietary intake, genetic predisposition, and the use of medicines that interfere with the folate-homocysteine cycle [6,7].

**Figure 1:**
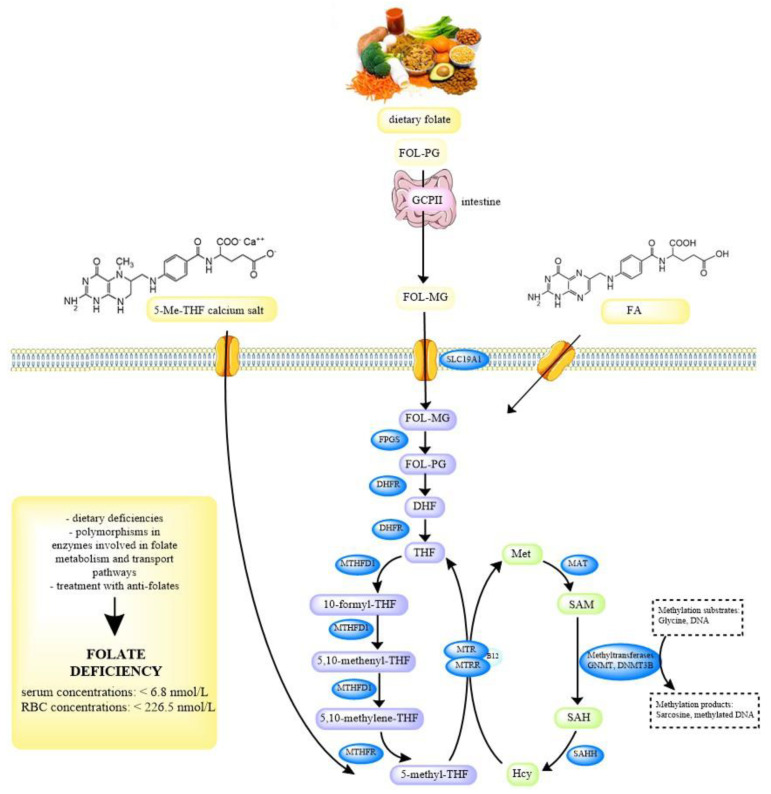
Overview of folate-homocysteine metabolism pathway and possible causes of folate deficiency. Humans can obtain folate from food or food supplements, FA and 5-Me-THF. Folates from food are polyglutamates and have to be hydrolized to monoglutamates in the intestine prior to absorption by the enzyme GCPII. FA and 5-Me-THF are absorbed directly without modification. Dietary folate and FA also need enzymatic activation; after entry through receptors in the cell membrane, folates are polyglutamated by FPGS and reduced to DHF and THF by DHFR. The latter enters the folate cycle (violet), which includes numerous other enzymes, MTHFD1, MTHFR, MTR, and MTRR, and is tightly connected to the methionine cycle (green), where homocysteine is re-methylated back to methionine. FOL-PG folate polyglutamate, GCPII glutamate carboxypeptidase II, FOL-MG folate monoglutamate, FA folic acid, SLC19A1 reduced folate carrier protein, FPGS folylpolyglutamate synthase, DHFR dihydrofolate reductase, DHF dihydrofolate, THF tetrahydrofolate, MTHFD1 methylenetetrahydrofolate dehydrogenase 1, MTHFR 5,10-methylenetetrahydrofolate reductase, MTRR methionine synthase reductase, MTR methionine synthase, B12 vitamin B_12_, Met methionine, MAT methionine adenosyltransferase, SAM S-adenosylmethionine, GNMT glycine *N*-methyltransferase, DNMT3B DNA methyltransferase 3 beta, SAH S-adenosylhomocysteine, SAHH S-adenosylhomocysteine hydrolase, Hcy homocysteine, RBC red blood cell.

Dietary folates predominantly exist as polyglutamates, which must be deconjugated to monoglutamates prior to absorption. This is accomplished by the enzyme glutamate carboxypeptidase II (GCPII) in the small intestine. Folate monoglutamates, including FA, 5-Me-THF, and 5-formyl-tetrahydrofolate are actively transported across the enterocytes by two transporters, the reduced folate carrier (RFC or SLC19A1) and the proton-coupled folate transporter (PCFT). The subsequent conversion of monoglutamates to polyglutamates ensures the retention of folate that would otherwise be lost by efflux from the cell. In order to enter the folate cycle, folate polyglutamates must first be reduced to dihydrofolate (DHF). Next, they are converted to an active form, 5-methyltetrahydrofolate (5-Me-THF), which is the main folate metabolite in the blood, by the 5,10-methylenetetrahydrofolate reductase (MTHFR). This enzyme is a key enzyme in the folate and homocysteine metabolism. MTHFR catalyzes the reduction of 5,10-methylenetetrahydrofolate to 5-Me-THF, which provides the methyl group for the remethylation of homocysteine to methionine, during which tetrahydrofolate (THF) is formed. Methionine can then be converted to S-adenosylmethionine (SAM), which is a main methyl donor in numerous reactions, including the methylation of DNA, proteins, phospholipids, neurotransmitters, and other small molecules. The intracellular folate-homocysteine metabolism is shown in Figure 1 [8,9,10].

In addition to dietary intake, plasma levels of folates are also modulated by genetic predisposition. Polymorphisms in genes coding for enzymes of the folate cycle are common—many of them with over 25% minor allele frequency (MAF) in the general population. The most critical are polymorphisms of the *MTHFR (5,10-methylenetetrahydrofolate reductase)* gene. MTHFR catalyses the conversion of 5,10-methylene-THF to biologically active folate, 5-Me-THF in an irreversible reaction [11]. The most important genetic polymorphisms that decrease the activity of MTHFR are two common functional polymorphisms, 677C>T (rs1801133) and 1298A>C (rs1801131), which are associated with reduced plasma levels of biologically active folate [12,13]. Other polymorphisms in genes involved in folate uptake and metabolism could also affect the folate-homocysteine pathways and alter folate and/or homocysteine levels, including *MTHFD1* 1958G>A (rs2236225) [14], *SLC19A1* 80A>G (rs1051266) [15], *MTRR* 66A>G (rs1801394) [9], and others. Several studies report that individuals carrying these SNPs had altered levels of folate and/or homocysteine, although the results vary in the literature [16,17,18].

Next, the folate levels depend on the use of medicines that interfere with the folate-homocysteine-methionine cycle. Numerous medicines disturb folate metabolism by a variety of mechanisms—they inhibit different enzymes of the folate cycle, such as DHFR and MTHFR, impair folate and vitamin B_12_ absorption, alter elimination of folate, induce liver enzymes, and act in numerous other ways. Examples of these medicines include trimethoprim, methotrexate, valproic acid, sulfasalazine, phenobarbital, phenytoin, and metformin [3].

To accurately evaluate folate status, monitoring must include total plasma and red blood cell (RBC) folate concentrations, which serve as sensitive biomarkers. Plasma folate is an indicator of recent folate intake, and intracellular folate concentration in RBC is an indicator of long-term folate status, influenced by folate availability in food and folate levels in blood. Plasma folate concentrations of less than 6.8 nmol/L and RBC concentrations below 226.5 nmol/L indicate folate deficiency. The normal range of folate plasma levels, based on a report of a World Health Organization (WHO) scientific group, is 13–45 nmol/L; intermediate levels (6.8–13 nmol/L) represent possible deficiency [19]. Another sensitive, indirect marker of inadequate folate levels is plasma homocysteine concentration. Folate insufficiency decreases the ability to re-methylate cellular homocysteine due to an inadequate concentration of 5-Me-THF in which it leads to an increased level of homocysteine. Homocysteine levels could also increase in the case of cobalamin (vitamin B_12_) deficiency, since B_12_ is an essential cofactor in the re-methylation cycle that converts homocysteine to methionine [20].

An increased need for folates is linked to adolescence, pregnancy, lactation, malabsorptive disorders, and diabetes, as well as anti-folate therapies [1,2,21,22]. The WHO recommends a daily intake of 400 µg of folic acid (FA) for women of childbearing age, which should enable an optimal plasma folate concentration of 55 nM [23,24]. Exceptionally high doses of 1 to 5 mg of FA are prescribed for women at high risk of having a child with NTD, and in chronic conditions of the gastrointestinal tract [25,26]. Folate deficiency is a severe public health risk, and is linked to severe congenital malformations. In 1998, to combat this risk, the U.S. Food and Drug Administration (FDA) required the addition of folic acid to enriched grain products, such as bread, pasta, rice, and, cereal. This measure, as well as high-dose FA supplements prescribed to at-risk populations, have the risk of causing excessive FA intake, and have unfavourable development consequences, such as increased risk for autism spectrum disorders [27].

Limitations in the understanding of folate bioavailability make accurate interpretation of adequate levels difficult. The most commonly used folate supplement is folic acid (pteroylglutaminc acid; FA), a synthetic oxidized form of folate, which is more stable and more readily absorbed than naturally occurring folates. Approximately 10 years ago, a synthetic reduced form of folate, 5-methyltetrahydrofolate (5-Me-THF), was developed and approved as an alternative supplement [28,29]. Currently, there are no recommendations for daily supplementation with 5-Me-THF, although it has some advantages over FA; 5-Me-THF, being an active form of folate, does not need enzymatic conversion to enter the folate cycle. As such, it could reduce the risk of lower conversion of FA to its active form, caused by medicines that inhibit enzyme DHFR, such as methotrexate, trimethoprim, triamterene, and sulfasalazine [3,10,30].

Variation in response to folates as well as other xenobiotics is multifactorial, and includes genetic and environmental factors. Evaluation of genetic contribution typically comes from clinical observations, while the contribution of environmental factors is difficult to control and manipulate in the living environment of clinical subjects. These limitations can be overcome by applying cell-based pharmacogenomic models, such as the lymphoblastoid cell lines (LCLs). LCLs are generated from fresh lymphocytes isolated from blood samples donated by consenting healthy adults and transformed by the Epstein-Barr virus (EBV); therefore, they can be grown in culture. Their nuclear DNA remains intact after immortalization; consequently, LCLs are a preferred method of storing an individual’s genetic material in biobanking [31]. These cell lines display the full spectrum of enzymes of the folate-homocysteine metabolism pathway and their reactions, and are a suitable model for studying metabolites of the folate-homocysteine cycle. LCL models have been widely used for forward and reverse translational research, where phenotypic observation or clinical findings are validated by functional LCL follow-up studies aiming to understand the mechanism of action. LCLs provide a cost-effective testing system where environmental factors and dosage can be controlled. Using LCL derived from diverse individuals makes it possible to mimic population diversity.

The aim of our study was to investigate the molecular mechanism underlying folate deficiency by addressing the three main factors influencing folate levels: the forms and dosages of intake, and genetic predisposition. Specifically, we aimed to determine the effects of folate deprivation and the potency of FA or 5-Me-THF supplementation on the reversal of phenotype. Furthermore, we aimed to evaluate the efficacy of 5-Me-THF in overcoming genetic defects of MTHFR polymorphisms. We set up the contemporary translational pharmacogenomics approach, employing the EBV-transformed lymphoblastiod cell lines (LCLs) that have been widely used for both the forward as well as reverse translational research, where observation or clinical findings are validated by functional LCL follow-up studies. The translational aspect of the present study represents a good basis for future clinical investigations and implementation.

## 2. Materials and Methods

### 2.1. Chemicals

Folic acid (FA), calcium d,l-5-methyltetrahydrofolate (5-Me-THF), d,l-homocysteine (Hcy), and S-(5′-Adenosyl)-l-methionine *p*-toluenesulfonate salt (SAM) were purchased from Sigma-Aldrich (Darmstadt, Germany). For cell culture applications, stock solutions of FA (5 mg/mL in DMSO) and 5-Me-THF (5 mg/mL in dH_2_O) were diluted in cell culture medium to the appropriate concentration, prior to the addition to cells.

The isotopically labelled compounds used as internal standards were levomefolic acid-13C,d3 from Santa Cruz Biotechnology (Heidelberg, Germany) and d,l-homocysteine-d4 from Toronto Research Chemicals (North York, ON, Canada). HPLC-grade acetonitrile ChromasolV^®^, acetic acid SupraPur^®^, perchloric acid, ascorbic acid, citric acid monohydrate, and perchloric acid were from Sigma-Aldrich (Darmstadt, Germany). Methanol and 1,4-dithiotreitol were provided by Merck (Darmstadt, Germany) and ultra-pure water was produced by an ELGA PureLab Flex LabWater purification system (UK MilliQ water; A10 Advantage, Millipore, Burlington, MA, USA).

### 2.2. Cell Culture

Human lymphoblastoid cell line cells (LCLs) were a kind gift from the National Laboratory for the Genetics of the Israeli Population (NLGIP), a human diversity biobank at Tel Aviv University, Tel Aviv, Israel (*n* = 35). LCLs are generated from fresh lymphocytes isolated from blood samples donated by consenting healthy adults and transformed by the Epstein-Barr virus (EBV); therefore, they can be grown in culture. Besides that, their nuclear DNA remains intact after immortalization; consequently, LCLs are a preferred method of storing an individual’s genetic material in biobanking [31]. These cell lines display the full spectrum of enzymes of the folate-homocysteine metabolism pathway and their reactions, and are a great model for studying metabolites of the folate-homocysteine cycle. LCLs were cultured in a RPMI 1640 medium (R5886, Sigma-Aldrich, Darmstadt, Germany) supplemented with 10% foetal bovine serum (Thermo Fisher Scientific, Gibco, Waltham, MA, USA), 4 mM l-glutamine, 100 U/mL penicillin, and 100 µg/mL streptomycin (all from Sigma-Aldrich, Darmstadt, Germany). For the experiments, LCLs were cultured in a RPMI 1640 without folic acid (L0503, Biowest, Nuaillé, France) supplemented with the same concentrations of foetal bovine serum, l-glutamine, penicillin, and streptomycin as classical RPMI 1640. When folate-free media was supplemented with serum, detectable levels of FA, (approximately 0.6 nM) were determined. On the other hand, standard RPMI medium (R5886, Sigma-Aldrich, Darmstadt, Germany) contains approximately 2266 nM (1 mg/L) of FA, an almost 4000-fold higher concentration compared to folate-free medium.

Cells were maintained at a concentration of between 1 × 10^6^ and 2 × 10^6^ cells/mL and cultured in a humidified chamber at 37 °C and 5% CO_2_.

### 2.3. CFSE Staining Assay

Proliferation of LCL cells was assessed using the CellTrace^TM^ Cell Proliferation Kit (Invitrogen Molecular Probes, Carlsbad, CA, USA) following the manufacturer´s instructions. Cells were washed and re-suspended in PBS at 1.0 × 10^6^ cells/mL and incubated with CFSE (final concentration 5 µM) for 15 min at 37 °C. Next, cells were washed and re-suspended in fresh pre-warmed culture medium and incubated for 30 min at 37 °C for complete modification of the probe. After the final wash step, cells were re-suspended in culture medium at 3 × 10^5^ cells/mL and treated with 50 nM FA or 5-Me-THF for 0, 24, 48, and 72 h. After the indicated time points, samples were examined using flow cytometry (Attune NxT, Invitrogen, Carlsbad, CA, USA) and evaluated by FlowJo software.

### 2.4. Cell Cycle Analysis

The assay was performed according to the manufacturer’s instructions. Briefly, LCLs (1 × 10^6^) were cultivated in the classical RPMI 1640 medium or in a folate-deficient medium (L0503) for one week and then treated with the compound of interest for 72 h. The cells were then washed with PBS and subjected to fixation with absolute ethanol at −20 °C for 15 min. Fixated cells were pelleted by centrifugation and rehydrated in 1 mL PBS for 15 min at room temperature. Collected cells were resuspended in 500 µL staining buffer (3 µM propidium iodide, 100 mM Tris, pH 7.4, 150 mM NaCl, 1 mM CaCl_2_, 0.5 mM MgCl_2_, 0.1% Nonident P-40). After 15 min of incubation, samples were analysed using Attune NxT Flow Cytometer (Invitrogen, Carlsbad, CA, USA) and evaluated by FlowJo software.

### 2.5. Metabolic Activity Assay

Prior to each experiment, cells were counted and diluted to the respective density of 3.0 × 10^5^ cells/mL. The viability of cells was assessed by means of tetrazolium MTS assay using the CellTiter96^®^ Aqueous One Solution Cell Proliferation Assay (Promega GmbH, Walldorf, Germany) according to the manufacturer´s instructions. The cells were treated with appropriate amounts of compounds of interest or corresponding vehicle (vehicle control: DMSO (<0.01%) was the vehicle control for all FA additions, and water was the vehicle control for all 5-Me-THF additions). Assays were performed in triplicate in 96-well plates. Three hours before the indicated time points, 10 µL of MTS reagent was added to each well containing 100 µL of cell suspension. Absorbance at 492 nm was measured after 3 h incubation on an automated microplate reader, the Synergy™ HTX Multi-Mode Microplate Reader (BioTek Instruments, Inc., Winooski, VT, USA). The relative cell viability of treated cells was calculated by subtracting the negative control, which contained no cells, and normalized to vehicle-treated control cells.

### 2.6. DNA Extraction and Genotyping

DNA was extracted from LCLs using the conventional salting-out procedure by the MasterPure complete DNA and RNA purification kit (Epicentre, an Illumina company, CA, USA). Ten common polymorphisms (MAF ≥ 25%) in nine genes involved in folate uptake and metabolism were genotyped by means of TaqMan (Applied Biosystems, Foster City, CA, USA) or LightSNiP (TIB MOLBIOL, Germany) probes, in accordance with the manufacturers’ instructions. Genotyping of rs1051266 (SLC19A1) was done using LightSNiP, while genotyping of the following polymorphisms was performed using TaqMan probes: rs1544105 (FPGS), rs1677693 (DHFR), rs2236225 (MTHFD1), rs1801133 and rs1801131 (MTHFR), rs1801394 (MTRR), rs10948059 (GNMT), rs2424913 (DNMT3B), and rs3733890 (BHMT).

### 2.7. Intracellular Hcy, 5-Me-THF and SAM Levels

LCLs were cultured in a RPMI 1640 medium deprived from folic acid for one week and then exposed to either 50 nM FA or 5-Me-THF for 72 h.

For the determination of intracellular concentrations of Hcy and 5-Me-THF, 10 million cells were pelleted and washed with 1 × PBS (pH = 7.4), and 150 µL of MiliQ water-containing antioxidants (DTT, ascorbic acid, and citric acid) was added to the pellet of cells and further processes as required, or stored at −80 °C before analysis. Frozen cell lysates were then thawed, and the internal standards of Hcy (4050 ng/mL) and 5-Me-THF (3 ng/mL) were added to each sample. The mixture was vortexed and sonicated (15 s, 40 mA), and then 750 µL ice-cold acetonitrile was added to each sample, vigorously vortexed (30 s), and left to stand on ice for approximately 20 min to allow protein precipitation. After that, the samples were centrifuged at 10,000× *g* for 10 min at 4 °C. The supernatants were transferred into new Eppendorf tubes and dried under a gentle stream of nitrogen at 40 °C. The residue was dissolved in 100 µL aliquots of MeOH:water (1:10), containing 100 µg/mL ascorbic acid, 100 µg/L citric acid, and 15 mg/mL DTT. The reconstituted sample was transferred to an autosampler vial with insert and subjected to LC-MS/MS analysis. The Agilent 1290 Infinity liquid chromatography system (Agilent Technologies, Santa Clara, CA, USA) and 6460 Triple Quad Mass Spectrometer (Agilent Technologies, Santa Clara, CA, USA) was used to analyze the samples of cell lysates. Chromatography separation was achieved on a Kinetex C18 column (100 × 3.0 mm, 2.6 µm) with a total run time of 12.10 min using a linear gradient of 0.05% acetic acid in water [32].

For determination of intracellular levels of SAM, other method and a different sample preparation procedure were used. Five million treated cells were pelleted and washed with 1 × PBS (pH = 7.4) and stored at −80 °C until analysis. Frozen cell lysates were then thawed and lysed in 0.5 M perchloric acid for 20 min on ice. After centrifugation (14,000× *g*, 15 min, 4 °C), supernatants were transferred to an autosampler vial with insert and measured by the reversed-phase high-performance liquid chromatography (HPLC) method with UV detection, as described previously [33].

The concentration of all analytes, Hcy, 5-Me-THF, and SAM were reported as the amount (ng for Hcy, 5-Me-THF, and nmol for SAM) per mg of protein. Protein concentrations were determined with the BioRad Assay Kit (Hercules, CA, USA) according to the manufacturer´s instructions.

### 2.8. Statistical Analyses

All the experiments were performed in a minimum of two individual biological replicates. All the MTS assays and DC protein assays were performed in triplicate. The data are expressed as the average ± SD. The normal distribution of data was checked by the D’Agostino and Pearson omnibus normality test and Shapiro-Wilk normality test. One-way ANOVA with post hoc Tukey’s multiple comparisons test was used to compare the percentage of cells in the S-phase, as well as Hcy, 5-Me-THF, and SAM levels in cells treated with different folate supplements: FA or 5-Me-THF calcium salt. Two-way ANOVA with Dunnett’s test for multiple comparisons was used to compare the MA of cells treated with different concentrations of folate supplement with control cells with no folates and Sidak’s multiple comparisons test to compare FA and 5-Me-THF treatment at each concentration. For all statistical analyses, GraphPad Prism Software was used. Means were considered statistically significant for *p* < 0.05, whereas *p*-values < 0.01, < 0.001, or < 0.0001 were considered as highly statistically significant.

## 3. Results

### 3.1. Folate Deficiency Causes an Arrest in the S-Phase of the Cell Cycle

LCLs are a well-established in vitro model of Epstein-Barr virus (EBV)-transformed B lymphocytes derived from healthy volunteers, and are used to test inter-individual responses due to a diverse genetic background.

**Figure 2:**
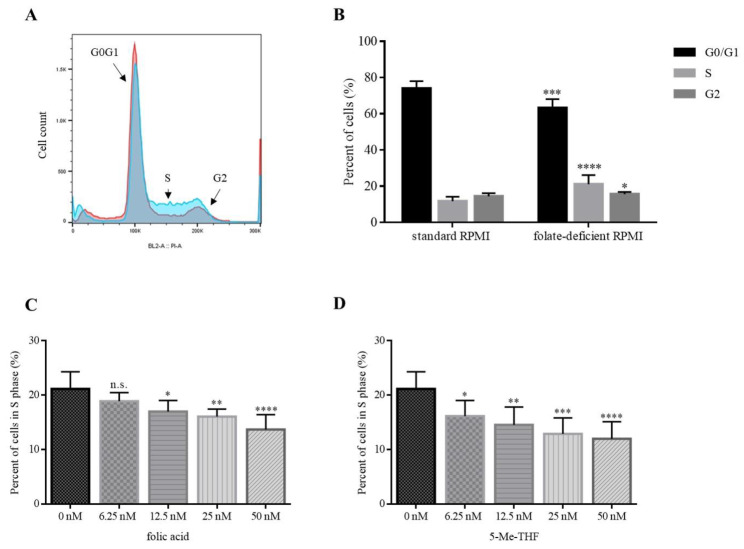
Folate deficiency causes a reversible arrest in the S-phase of the cell cycle. (**A**) Representative histogram of the cell cycle of LCL using alcohol fixation and propidium iodide DNA staining. LCLs were cultured for 10 days in standard RPMI medium (red curve) and in medium without FA (blue curve). (**B**) Graph represents the average proportion of cells in the G0/G1, S, and G2 phase of cells cultured in standard RPMI medium and in folate-deficient medium (*n* = 10). (**C**,**D**) Concentration-dependent reversal of the proportion of cells in the S-phase. LCLs were cultured in folate-deficient medium for one week and then cultured for 72 h in the presence of different concentrations (6.25 nM, 12.5 nM, 25 nM and 50 nM) of FA (**C**) or 5-Me-THF (**D**). In each experiment, 1 × 10^6^ cells of each LCL were analysed. Values are means ± SD. Statistical differences were determined by ANOVA with Tukey’s multiple comparison test. n.s. (not significant) *p* > 0.05, * *p* ≤ 0.05, ** *p* ≤ 0.01, *** *p* ≤ 0.001, **** *p* < 0.0001 compared to control (*n* = 10).

To evaluate the effects of folate on cell proliferation, we used 10 different LCLs and cultured them either in folate-free or in complete growth media. A representative histogram is shown in Figure 2A.

When cultured in standard complete RPMI medium, the proportion of cells in the S-phase of the cell cycle was 11.7%. After 10 days of cell culturing in the folate-deficient medium, the proportion of cells in the S-phase of the cell cycle increased to 21.1% (Figure 2B). This indicates that folate deficiency causes an arrest in the S-phase (*p* ≤ 0.0001). Accumulation of cells in the S-phase was accompanied with a decrease in the G0/G1 phase (74.0% versus 63.2%; *p* ≤ 0.001) and a slight, but still significant increase in the G2 phase (14.3% versus 15.7%; *p* ≤ 0.05) (Figure 2B).

To evaluate whether folate-deficiency-induced S-phase arrest is reversible, we used folic acid (FA) and 5-methyltetrahydrofolate (5-Me-THF) as supplements. We showed that folate supplementation rescues cells from S-phase arrest in a concentration-dependent manner.

When 6.25 nM, 12.5 nM, 25 nM, and 50 nM 5-Me-THF were added into folate-deficient medium, the proportion of cells in the S-phase decreased to 16.1% (*p* = 0.038), 14.5% (*p* = 0.0032), 12.9% (*p* = 0.0002), and 12.0% (*p* < 0.0001), respectively (Figure 2D). When the equimolar concentrations of FA were added, the proportions of cells in the S-phase were 18.9% (n.s.; *p* = 0.38), 17.0% (*p* = 0.024), 16.1% (*p* = 0.0042), and 13.7% (*p* < 0.0001), respectively (Figure 2C).

Both agents were able to reverse the accumulation of cells in the S-phase. When using physiologically relevant concentrations (50 nM) of FA or 5-Me-THF, the difference between the two was not statistically significant (*p* = 0.19). The proportion of cells in the S-phase was comparable to those cultured in complete media (50 nM 5-Me-THF: 12.0%, standard RPMI: 11.7%).

### 3.2. Folate Supplements Increase the Metabolic Activity of Cells

Next, we examined whether the metabolic activity (MA) of the cells depended on the type of folate supplement.

**Figure 3:**
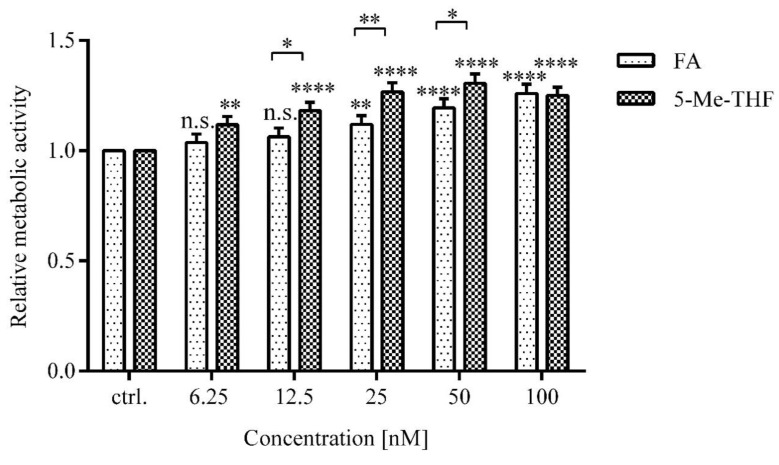
Concentration-dependent effect of FA and 5-Me-THF on metabolic activity of LCLs. Folate-deprived LCLs (*n* = 35) were incubated with five different concentrations of FA and 5-Me-THF for 72 h. Metabolic activity was assessed by an MTS assay. The response is expressed as fold change over vehicle-treated control. DMSO (<0.01%) was the vehicle control for all FA additions and water was the vehicle control for all 5-Me-THF additions. Values are means ± SD. Statistical differences were determined by two-way ANOVA with a post hoc Dunnett’s multiple comparisons test. n.s. (not significant) *p* > 0.05, * *p* ≤ 0.05, ** *p* < 0.01, **** *p* < 0.0001 compared to untreated control.

Higher MA was observed in LCLs treated with higher versus lower concentrations of folates (*p* < 0.0001) and in cells treated with biologically active folate (5-Me-THF) versus FA (*p* < 0.0001) (Figure 3).

The differences in MA of cells treated with 5-Me-THF compared to FA at 12.5 nM, 25 nM, and 50 nM were 12% (*p* = 0.031), 15% (*p* = 0.0071), and 11% (*p* = 0.048), respectively. At 6.25 nM and 100 nM, the difference between MA of LCLs treated with FA or 5-Me-THF was not evident (*p* = 0.18, *p* = 0.97).

MA increase was concentration-dependent at concentrations higher than 6.25 nM, representing folate deficiency states, and also up to 50 nM, which represents physiologically relevant concentration.

### 3.3. 5-Me-THF Is More Effective than FA in Increasing Metabolic Activity of Cells with Low MTHFR Activity

Folic acid has a complex metabolism, and its effects depend on the concentration of the biologically active form, 5-Me-THF. Enzyme 5,10-methylenetetrahydrofolate reductase (MTHFR) is the key folate pathway enzyme involved in the formation of this biologically active form, and it is very polymorphic; approximately 90% of the European population has polymorphic forms of MTHFR. The two most common genetic polymorphisms shown to decrease the activity of MTHFR and influence the levels of biologically active folate are 677C>T (rs1801133) and 1298A>C (rs1801131) [11]. To investigate whether these two polymorphisms modify cell response to FA or 5-Me-THF, LCLs were genotyped for the polymorphisms rs1801133 and rs1801131 using TaqMan probes.

LCLs selected for further experiments were of diverse genotypes—five LCLs were determined as wild-type homozygous for both genotypes (677CC/1298AA), while others were determined as heterozygotes; nine LCLs carried the 677CT/1298AA, five LCLs 677TT/1298AA, four LCLs 677CC/1298AC, eleven LCLs 677 CT/1298AC, and one LCL carried the 677CC/1298CC genotype. However, no LCLs carried the 677CT/1298CC, 677TT/1298AC, or 677TT/1298CC genotypes (Table 1). These three genotype combinations were reported to be extremely rare in the general population, since rs1801133 and rs1801131 are usually in *trans* and very rarely in *cis* configurations, indicating that these two polymorphisms are in incomplete linkage disequilibrium [34].

The next important determinant factor of intracellular folate availability is the reduced folate carrier (RFC), the principal transporter by which folates enter the cell. The common SNP SLC19A1 c.80A>G (rs1051266) affects folate binding and uptake [35]. We genotyped LCLs for SLC19A1 c.80A>G using the LightSNiP probe; ten LCLs were non-mutated homozygotes (»wild-type«; GG), seventeen LCLs were heterozygotes (GA), and seven of them were mutated homozygotes (AA). The genotyping for SLC19A1 was not successful for one LCL.

To evaluate the effect of FA and 5-Me-THF on cells with diverse MTHFR activity, LCLs were segregated into two groups according to the predicted MTHFR activity based on the MTHFR genotype: (1) the normal MTHFR activity cell (>60% enzyme activity) group, which included genotype combinations 677CC/1298AA, 677CT/1298AA, 677CC/1298AC, and 677CC/1298CC; and (2) the low MTHFR activity cell (≤60% enzyme activity) group, which included genotype combinations 677CT/1298AC and 677TT/1298AA. To avoid the effect of RFC, cells with the mutated form of RFC were excluded from further analysis.

**Figure 4:**
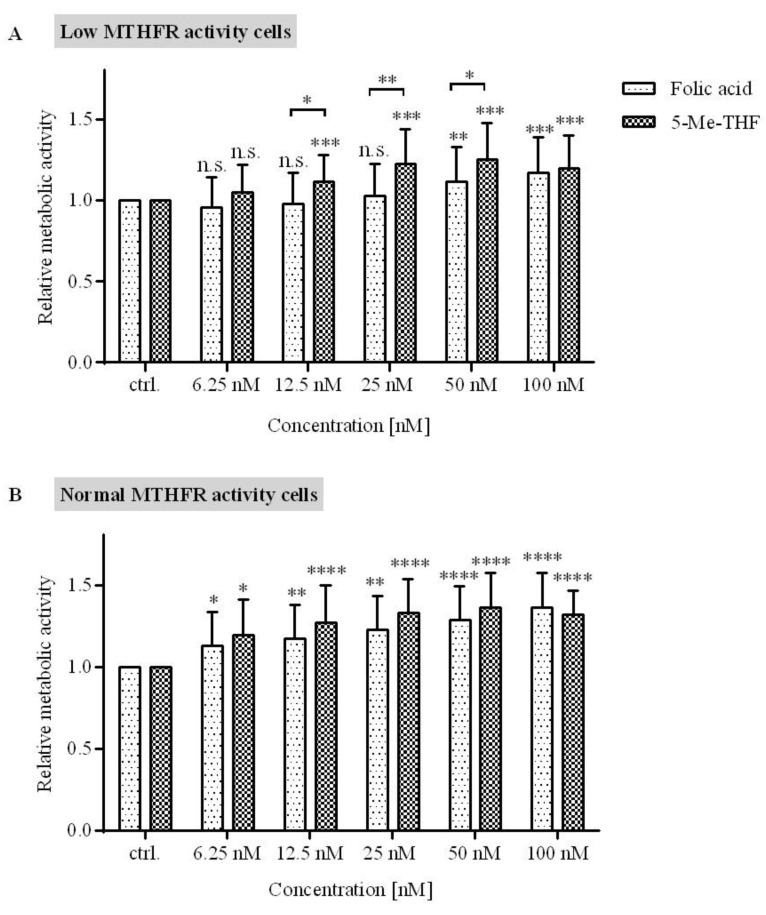
The influence of folate supplement type on the metabolic activity in LCLs with diverse MTHFR enzyme activity. (**A**) Cell lines with predicted low MTHFR activity (*n* = 15). (**B**) Cell lines with predicted normal MTHFR activity (*n* = 12). LCLs were maintained in folate-deprived media and then treated with five different concentrations of FA and 5-Me-THF for 72 h. Metabolic activity was assessed by an MTS assay. The response is expressed as fold change over vehicle-treated control. Values are means ± SD. Statistical differences were determined by two-way ANOVA with a post hoc Dunnett’s multiple comparisons test. n.s. (not significant) *p* > 0.05, * *p* ≤ 0.05, ** *p* ≤ 0.01, *** *p* < 0.0001, **** *p* ≤ 0.0001, compared to control.

The results showed that in cells with low MTHFR activity (≤60%), 5-Me-THF is more effective in increasing metabolic activity then FA (Figure 4A) at concentrations higher than 6.25 nM and up to 50 nM. The difference in metabolic activity between cells treated with 5-Me-THF and FA at a concentration of 12.5 nM was 14% (*p* = 0.045); at 25 nM, the difference was 20% (*p* = 0.0098); and at 50 nM, the difference was 14% (*p* = 0.047). At 100 nM concentration, this trend was lost (*p* = 0.70).

Cells with normal MTHFR enzyme activity are able to normally metabolize FA; therefore, it was not surprising that the metabolic activity was not different between cells treated with equimolar concentrations of either 5-Me-THF or FA (Figure 4B).

The effect of other polymorphisms in folate-metabolizing genes was also determined. All 35 LCL cells were genotyped for the following polymorphisms: rs105266 (SLC19A1), rs1544105 (FPGS), rs1677693 (DHFR), rs2236225 (MTHFD1), rs1801133 and rs1801131 (MTHFR), rs1801394 (MTRR), rs3733890 (BHMT), rs10948059 (GNMT), and rs2424913 (DNMT3B). However, we could not demonstrate any significant association of the analysed SNPs to metabolic activity or to the intracellular concentrations of folate analytes after folate supplementation. In order to detect any potential association, a larger sample of LCLs would be needed (Supplementary Table S1).

### 3.4. MTHFR Deficiency Impacts Intracellular Concentration of 5-Me-THF

The main folate metabolite is 5-Me-THF, which functions as a methyl donor for remethylation of homocysteine, leading to formation of methionine and THF. Another important metabolite in the methionine cycle is S-adenoslymethionine (SAM), the universal methyl donor. The levels of Hcy and SAM vary with folate level. Plasma Hcy, SAM, and folate are widely used as indicators of folate sufficiency, methylation capacity, and risk for heart diseases and cancer. Although plasma metabolites are well-established biomarkers of disease, an understanding of the mechanism of disease requires an understanding of intracellular metabolite levels.

There is increasing evidence suggesting that polymorphisms in the *MTHFR* gene influence the plasma levels of biologically active folate [11,36,37]. In order to investigate whether the two most common genetic polymorphisms of MTHFR, 677C>A and 1298A>T, influenced the intracellular concentration of biologically active folate, cells were divided into two groups, normal and low MTHFR activity groups based on the expected enzyme activity (>60% or ≤60%). Cells with mutated reduced folate carrier (SLC19A1 genotype AA) were excluded from the analysis.

**Figure 5:**
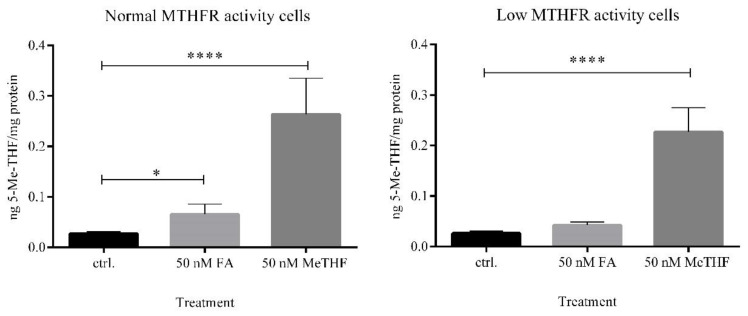
Intracellular 5-methyltetrahydrofolate concentrations. LCLs were treated with equimolar concentration of folate supplements, FA and 5-Me-THF, for 72 h. The concentration of 5-Me-THF was measured in cell lysates with LC-MS/MS. Cells with a defective reduced folate carrier were excluded from the analysis. Statistical differences were determined by one-way ANOVA with a post hoc Tukey’s multiple comparisons test. Values are means ± SD. * *p* ≤ 0.05, **** *p* ≤ 0.0001, compared to untreated control (ctrl.).

First, intracellular concentrations of 5-Me-THF were investigated using LC-MS/MS. When cells with normal MTHFR activity were exposed to FA, the intracellular concentration of 5-Me-THF was 2.5-fold higher compared to the untreated control (*p* = 0.013). In contrast, when cells with low MTHFR activity were exposed to FA, we did not observe statistically significant differences in the intracellular level of 5-Me-THF (*p* = 0.10) (Figure 5). Even though cells with low MTHFR activity were divided into the group of compound heterozygotes (677CT/1298AC) and into the group of homozygotes 677TT, the addition of FA into media had no significant effect on intracellular concentrations of 5-Me-THF (Supplementary Figure S1).

These data showed that the concentration of a biologically active form of 5-Me-THF after FA supplementation is dependent on MTHFR genotype.

Next, when cells were exposed to 50 nM 5-Me-THF, high intracellular concentrations of the same analyte were observed in both subgroups. This finding indicates the expected transport of 5-Me-THF supplement through a cell membrane into the cells.

We also evaluated the effect of folate supplements on the intracellular concentrations of SAM and Hcy. Quantification of Hcy was performed with the same LC-MS/MS method as it was for the analyte 5-Me-THF, while the quantification of SAM was performed with the HPLC-UV/vis method. When cells were treated with 50 nM FA or 5-Me-THF, intracellular concentration of SAM was 1.3-fold higher than it was in the untreated control group; it increased from 0.829 ± 0.180 nmol SAM/mg protein to 1.080 ± 0.263 nmol SAM/mg protein (*p* < 0.0001) or to 1.052 ± 0.213 nmol SAM/mg protein (*p* < 0.0001) (Supplementary Figure S2). In our experiments, which were carried out using medium with excess vitamin B_12_, we observed a slight but non-significant decrease of intracellular Hcy after the addition of folate supplementation.

No other significant differences in intracellular concentrations of SAM and Hcy, resulting from the MTHFR genotype, were determined (Supplementary Figures S3 and S4).

## 4. Discussion

Adequate folate levels are critical to cell homeostasis supporting normal embryonic development, and both deficiency and excess of folates possess health-related hazards [1,2,4]. Therefore, it is important to understand the bioactivation pathways of folates and the molecular mechanism underlying folate deficiency. We employed the EBV-transformed lymphoblastoid cell lines, which have proven to be a powerful tool in pharmacogenomics studies for assessing interindividual differences in response to xenobiotics. The aim of the current study was to evaluate the effect of folate deprivation and supplementation on cell growth and proliferation of human lymphoblastoid cells and to evaluate the potency of FA or 5-Me-THF supplementation on increasing intracellular concentration of biologically active folate in genetically diverse cells. FA and 5-Me-THF are the most commonly available folate supplements worldwide [38], but there is limited research on their bioavailability, their effects on growth and proliferation of the cells, and on their influence on intracellular concentrations of key folate-homocysteine metabolites.

First, we investigated how folate deficiency alters the cell cycle in vitro. Cells incubated in standard commercial cell culture medium are exposed to relatively high folate levels, up to 2 µM [39,40]. This is much higher compared to the normal range observed for folate in human plasma (13–45 nM) [19,41]. We used folate-deficient medium (RPMI 1640, L0503, Biowest, Nuaillé, France), which contained only FA present in dialyzed serum at the approximate concentration of 0.6 nM. Our results showed that the lack of folate caused an arrest in the S-phase of the cell cycle in LCLs cultured in folate-deficient medium for 10 days. The effect of folate deficiency on cell proliferation and on programmed cell death have been researched on many different cell models, including neural stem cells [42], erythroblasts [43], colonocytes [40], and also lymphocytes [44]. Our results are in agreement with those from Courtemanche et al., who showed that folate deficiency in normal human T lymphocytes induces arrest in the S-phase of the cell cycle [44]; however, Yang et al. showed that folate deprivation induces arrest in the G0/G1 phase in mouse hippocampal neuron cells (HT-22) [45].

Next, we showed a negative correlation between the extent of S-phase arrest and folate concentration and reversal of the observed phenotype upon supplementing with either FA or 5-Me-THF (Figure 2). The fact that folate repletion restored the proliferation of folate-deficient cells suggests that folate-deficient lymphocytes have an impaired capacity to synthesize DNA and to complete the cell cycle.

FA has a complex metabolism and its effects depend on the concentration of the biologically active form, 5-Me-THF [41]. Higher metabolic activity was observed in LCLs treated with higher versus lower concentrations of folates and in cells treated with biologically active folate (5-Me-THF) versus FA. Our results indicate that 5-Me-THF is more effective at increasing cell metabolic activity than FA.

Numerous clinical studies demonstrated a significant association between MTHFR polymorphisms and various diseases, such as serious congenital birth defects (orofacial clefts, congenital heart defects, neural tube defects), cardiovascular diseases, neuronal development diseases, cancers, and also psychiatric disorders [1,2,4,5]. The *MTHFR* gene is very polymorphic; the most studied polymorphisms that decrease the activity of MTHFR are 677C>T (rs1801133) and 1298A>C (rs1801131) [11]. Two groups of MTHFR activity were formed following the example of previous published studies that showed that compound heterozygosity for 677CT/1298AC will have similar clinical impact as C677T homozygosity [46,47]. The results showed that the low MTHFR activity group profited from 5-Me-THF supplementation. Such results were expected, since 5-Me-THF is independent of MTHFR capacity to actively metabolize folate. However, a high concentration of FA could potentially bypass conversion capacity resulting from polymorphisms in *MTHFR*. Our in vitro observations are in line with recommendations of the International Federation of Obstetrics and Gynecology (FIGO), which suggests an increase in the dosage of FA if there are polymorphisms in folate cycle genes [48].

Even though FA supplementation to supra-physiological levels has demonstrated many benefits to pregnant women, high-dose folate supplementation carries potential risks. First, folate supplementation can mask vitamin B_12_ deficiency and could cause serious neurological effects [49]. High folate may also contribute to insulin resistance in offspring, a higher risk of small size for gestational age infants, as well as higher risk for some cancers, depression, and cognitive impairment [4,5,50,51]. Furthermore, excessive FA could interact with medicines that inhibit the DHFR enzyme [3,10,30]. With all these concerns in mind, folate supplementation needs good monitoring.

Next, we investigated the differences between the effect of FA and 5-Me-THF supplementation on the intracellular concentrations of key folate metabolites, 5-Me-THF and Hcy. Folate-depleted LCLs were supplemented with 50 nM folate. The concentration was selected based on studies involving women of childbearing age. These studies reported mean plasma folate levels of 51 nM (administered dose 0.4 mg/d), 53–55 nM (administered dose 0.4 mg/d), and 44 nM (administered dose 0.375 mg/d) after 12 weeks of FA consumption [23,24,52]. The results of the present study show that the intracellular concentration of biologically active folate increased after the addition of either folate supplement; however, the addition of 5-Me-THF is more effective than the addition of FA.

Since polymorphisms in the *MTHFR* gene influence the levels of biologically active folate, we analyzed intracellular levels of 5-Me-THF after folate supplementation in LCLs with diverse MTHFR activity [11,36,37]. In the group with normal MTHFR activity cells, FA treatment significantly increased the concentration of biologically active folate, while in the group with low MTHFR activity cells, the change was not statistically different compared to untreated control cells. When cells were exposed to the equimolar concentration of 5-Me-THF, high intracellular concentrations of the biologically active form were determined in both subgroups. These results demonstrate that polymorphisms influencing the enzyme activity of MTHFR are associated with intracellular concentrations of the methylated form of folates.

On the other hand, when LCLs were exposed to FA or 5-Me-THF, intracellular levels of SAM increased independently of folate type or MTHFR enzyme activity. This is consistent with other studies which have investigated the relationship between MTHFR genotype and SAM levels, showing no association between the two [11,53,54].

Further, folate supplementation of LCLs appears not to influence the concentration of intracellular Hcy, despite the fact that folate supplementation is expected to decrease Hcy plasma levels in vivo [55,56]. Results obtained for intracellular Hcy and SAM concentrations do not follow expectations based on understanding of in vivo biochemical reactions and biological distribution in tissues, organs, and fluids. Our study is, however, in line with findings of a Dutch research group, which reported the response of intracellular concentrations of Hcy, SAM, and SAH to FA administrations in samples obtained from a randomized trial, and also found that FA supplementation did not reduce intracellular Hcy [57].

In this study, we used 35 LCLs derived from different individuals representing a diverse genetic background and genotyped them for 10 common polymorphisms (MAF ≥ 25%) in 9 genes involved in folate uptake and metabolism (SLC19A1, FPGS, DHFR, MTHFR, MTRR, MTHFD1, BHMT, GNMT, and DNMT3B).

As shown above, polymorphisms in *MTHFR*, which were analyzed together as genotype combinations and segregated into two groups according to the predicted MHTFR activity, correlated with the metabolic activity or with the intracellular concentrations of folate analytes. However, none of the other analysed polymorphisms correlated or showed statistical significance.

This study was highly focused and demonstrated a strong proof of concept which can form the basis of further investigation; however, the applicability of this study is limited by an experimental setup.

## 5. Conclusions

This study represents a translational pharmacogenetics approach employing lymphoblastoid cell lines (LCLs), where clinical observations of the importance of folates were revisited in vitro in order to understand the mechanism of action correlated to genetic determinants.

The eminent role of folates in cell division is demonstrated by reversible S-phase arrest in cells deprived of folates. The cells cultured in folate-deprived media had lower metabolic activity and slower propagation when compared to controls propagated in media supplemented with 50 nM folates.

Next, we provide evidence that 5-Me-THF can bypass folate insufficiency due to MTHFR deficiency. This finding is of immense importance, since a significant percentage of the population is unable to properly metabolize folate due to defects in the MTHFR enzymes they carry [58]. In such cases, folate supplementation with 5-Me-THF is more efficient than supplementation with FA, since it can enter the folate cycle directly, without the need for enzymatic modification [1,30] and could overcome metabolic defects. Nevertheless, the current in vitro LCL model has its limitations, including lymphoid tissue specificity and biological simplicity. Because of potential non-genetic and whole organism-based confounders, LCL studies must be replicated in clinical settings with follow-up functional validation studies.

These findings serve as a basis for further model development for preclinical testing and biomarker development, including folate supplementation and anti-folate therapies.

## Figures and Tables

**Table 1:** Number of LCL cells according to MTHFR C677T and A1298C genotypes.

Genotype		Non-Mutated Homozygote (»Wild-Type«)	Heterozygote	Mutated Homozygote
		677CC	677CT	677TT
**Non-mutated homozygote (»wild-type«)**	**1298AA**	5	9	5
**Heterozygote**	**1298AC**	4	11	**0**
**Mutated homozygote**	**1298CC**	1	**0**	**0**

## Supplementary Materials

The following are available online at https://www.mdpi.com/2077-0383/9/9/2836/s1, Figure S1: Intracellular 5-Me-THF concentrations in (A) NORMAL MTHFR activity cells (genotype 677CC/1298AA, 677CT/1298AA, 677CC/1298AC, and 677CC/1298CC) (*n* = 12) and in (B) LOW MTHFR activity cells (genotype 677TT/1298AA and 677CT/1298AC) (*n* = 15). (B.1) LCLs with 677TT/1298AA genotype (*n* = 5) and (B.2) LCLs with 677CT/1298AC genotype (*n* = 10)., Figure S2: Intracellular SAM concentration in folate-depleted cells (ctrl.) and after FA or 5-Me-THF treatment in LCLs., Figure S3: Intracellular SAM concentrations in (A) NORMAL MTHFR activity cells (genotype 677CC/1298AA, 677CT/1298AA, 677CC/1298AC, and 677CC/1298CC) (*n* = 12) and in (B) LOW MTHFR activity cells (genotype 677TT/1298AA and 677CT/1298AC) (*n* = 15). (B.1) LCLs with 677TT/1298AA genotype (*n* = 5) and (B.2) LCLs with 677CT/1298AC genotype (*n* = 10)., Figure S4: Intracellular Hcy concentrations in (A) NORMAL MTHFR activity cells (genotype 677CC/1298AA, 677CT/1298AA, 677CC/1298AC, and 677CC/1298CC) (*n* = 12) and in (B) LOW MTHFR activity cells (genotype 677TT/1298AA and 677CT/1298AC) (*n* = 15). (B.1) LCLs with 677TT/1298AA genotype (*n* = 5) and (B.2) LCLs with 677CT/1298AC genotype (*n* = 10)., Table S1: Genotype of the ten common polymorphisms of genes involved in folate uptake and metabolism in LCL cells.
