# Supplementary material for: Folate Insufficiency Due to MTHFR Deficiency Is Bypassed by 5-Methyltetrahydrofolate
Source: J Clin Med. 2020 Sep 2;9(9):2836. doi: 10.3390/jcm9092836 (PMC7564482; doi:10.3390/jcm9092836)
Supplement: Supplementary file 1 [file jcm-09-02836-s001.pdf]

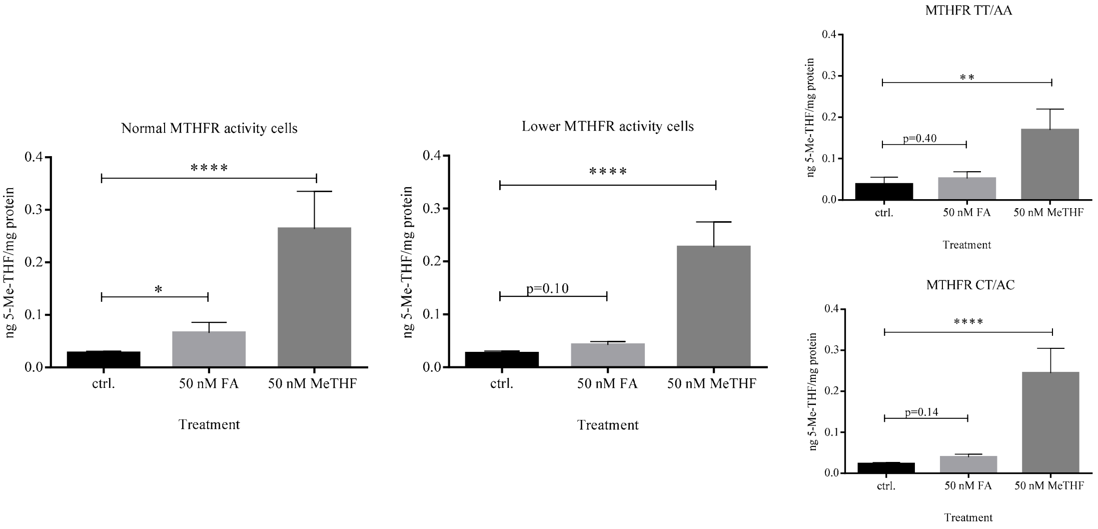

Supplementary Figure 1: Intracellular 5-Me-THF concentrations in (A) NORMAL MTHFR activity cells (genotype 677CC/1298AA, 677CT/1298AA, 677CC/1298AC, and 677CC/1298CC) (N=12) and in (B) LOW MTHFR activity cells (genotype 677TT/1298AA and 677CT/1298AC) (N=15). (B.1) LCLs with 677TT/1298AA genotype (N=5) and (B.2) LCLs with 677CT/1298AC genotype (N=10).

Statistical differences were determined by one-way ANOVA with post-hoc Tukey's multiple comparisons test. Values are means ± SD. * $p \leq 0.05$, ** $p \leq 0.01$, **** $p \leq 0.0001$, compared to untreated control (ctrl.).

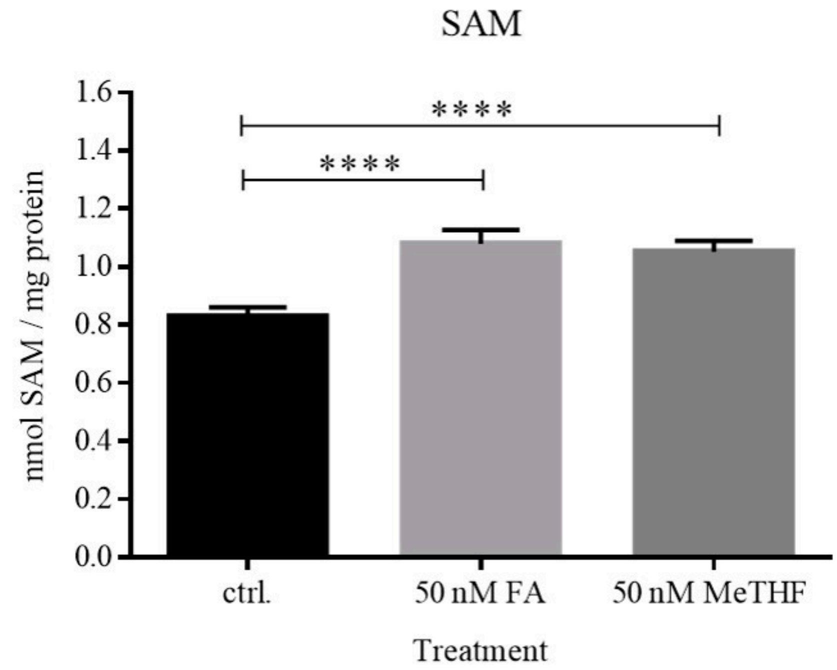

Intracellular concentration of SAM [ngmol SAM / mg protein]		
**Ctrl. (mean ± SD)**	**50 nM FA (mean ± SD)**	**50 nM 5-Me-THF (mean ± SD)**
0.829 ± 0.180	1.080 ± 0.263	1.052 ± 0.213

.

Supplementary Figure 2: Intracellular SAM concentration in folate-depleted cells (ctrl.) and after FA or 5-Me-THF treatment in LCLs. LCLs (N=35) were treated with equimolar concentration of folate supplements for 72h, then they were lysed and the concentration of SAM was measured with HPLC-UV/vis. Statistical differences were determined by one-way ANOVA with post-hoc Tukey's multiple comparisons test. Values are means ± SD. **** $p \leq 0.0001$, compared to control.

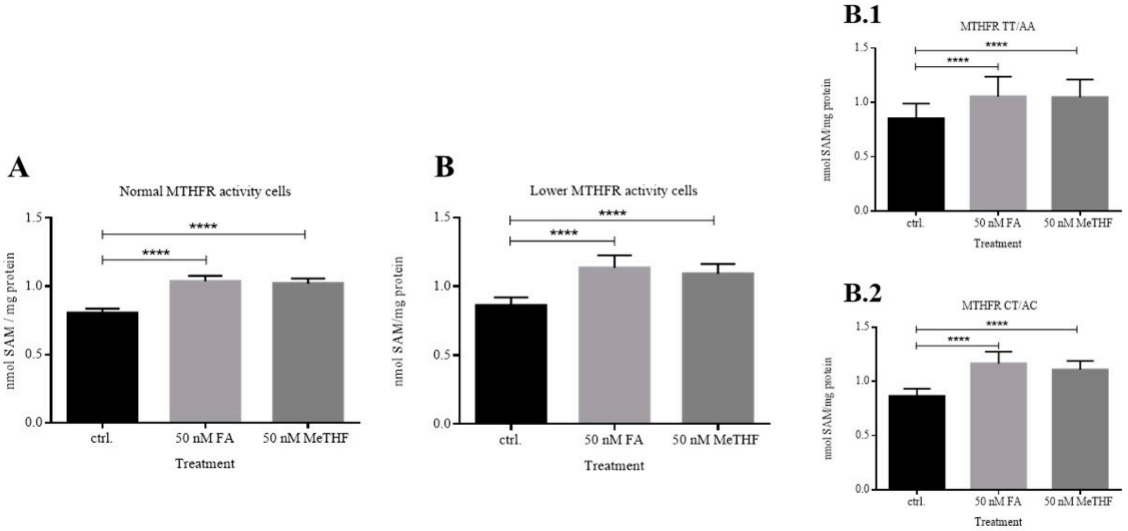

Supplementary Figure 3: Intracellular SAM concentrations in (A) NORMAL MTHFR activity cells (genotype 677CC/1298AA, 677CT/1298AA, 677CC/1298AC, and 677CC/1298CC) (N=12) and in (B) LOW MTHFR activity cells (genotype 677TT/1298AA and 677CT/1298AC) (N=15). (**B.1**) LCLs with 677TT/1298AA genotype (N=5) and (**B.2**) LCLs with 677CT/1298AC genotype (N=10).

Statistical differences were determined by one-way ANOVA with post-hoc Tukey's multiple comparisons test. Values are means ± SD. **** $p \leq 0.0001$, compared to untreated control (ctrl.).

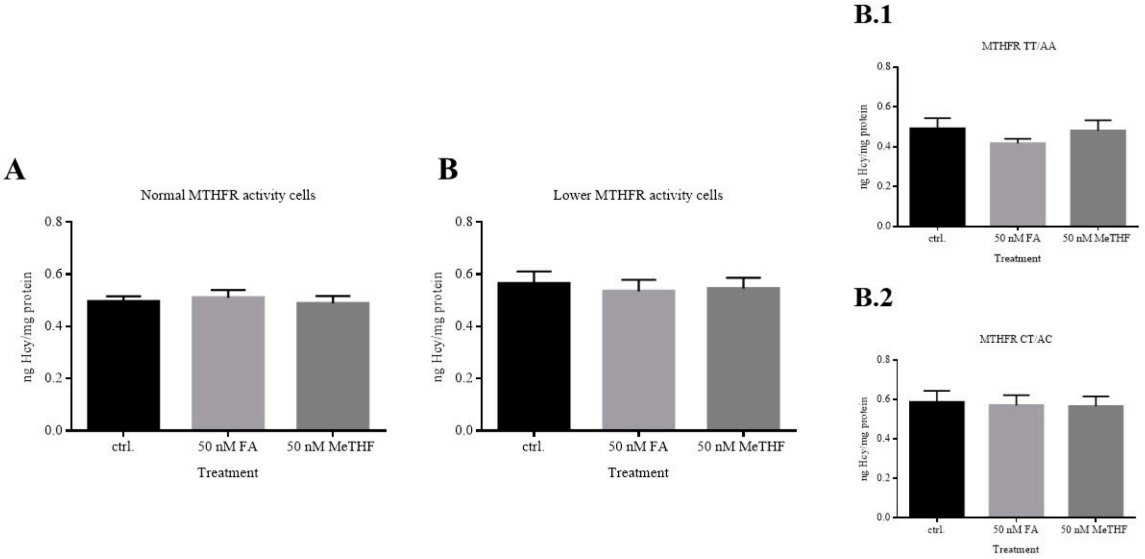

Supplementary Figure 4: Intracellular Hcy concentrations in (A) NORMAL MTHFR activity cells (genotype 677CC/1298AA, 677CT/1298AA, 677CC/1298AC, and 677CC/1298CC) (N=12) and in (B) LOW MTHFR activity cells (genotype 677TT/1298AA and 677CT/1298AC) (N=15). (**B.1**) LCLs with 677TT/1298AA genotype (N=5) and (**B.2**) LCLs with 677CT/1298AC genotype (N=10).

Statistical differences were determined by one-way ANOVA with post-hoc Tukey's multiple comparisons test. Values are means ± SD. There were no statistical significant changes on intracellular Hcy after the addition of folate supplements, compared to untreated control (ctrl.).

Supplementary Table 1: Genotype of the ten common polymorphisms of genes involved in folate uptake and metabolism in LCL cells.

	Gene (polymorphism)									
**ID** (LCL)	**SLC19A1** (rs105266)	**FPGS** (rs1544105)	**DHFR** (rs1677693)	**MTHFD1** (rs2236225)	**MTHFR 677** (rs1801133)	**MTHFR 1298** (rs1801131)	**MTRR** (rs1801394)	**BHMT** (rs3733890)	**GNMT** (rs10948059)	**DNMT3B** (rs2424913)
**1112**	GA	CT	CC	AA	TT	AA	AG	GG	CT	CC
**1122**	GA	CC	CA	GG	CC	AC	AG	GG	TT	CC
**1515**	GA	CT	CA	GA	CT	AC	AG	GA	TT	TT
**1516**	GG	TT	CA	AA	TT	AA	GG	GG	CC	CT
**1570**	AA	CT	CA	GG	CC	AC	AA	GA	CT	TT
**1728**	GA	CC	CC	GA	CT	AC	AA	GA	TT	CC
**1754**	AA	CC	CC	AA	CT	AA	AG	GG	CT	CT
**1801**	/	CC	CC	AA	CC	AA	AA	AA	CT	CC
**1823**	GG	CT	CC	GA	CT	AC	AG	GA	CC	CC
**1976**	GG	CT	CC	GG	CT	AC	AG	GG	CC	CT
**4973**	AA	CT	CC	GA	CT	AA	AG	GG	CC	CT
**4995**	AA	CT	CC	GG	CT	AA	AA	GG	CC	CT
**5876**	GG	CT	CC	AA	CT	AC	AA	GG	CT	TT
**6037**	GA	CC	CC	AA	CT	AA	AG	GG	TT	TT
**6127**	GA	CC	CA	GA	CT	AC	GG	GA	CC	CC
**6153**	GA	CC	CC	GA	CT	AC	AG	GA	CC	CC
**6159**	GG	CC	CC	AA	TT	AA	AG	GG	CC	CT
**6203**	GA	CT	CC	AA	CT	AA	AG	GG	CC	CT
**6333**	GA	CT	CC	AA	CT	AC	AG	GG	CT	CC
**6417**	GA	CT	CC	GG	CT	AA	AG	GG	CC	CC
**6423**	GG	CC	CA	AA	TT	AA	AA	GG	CT	CC
**6425**	AA	CT	AA	GA	TT	AA	GG	GG	CT	TT
**6429**	GG	CT	CC	GA	CT	AC	GG	GG	CC	TT
**6430**	GA	TT	CA	GA	CT	AC	GG	GA	CT	CC
**380**	AA	CC	CC	AA	CC	AA	AG	GG	CT	TT
**381**	AA	CT	CC	GA	CC	AA	AG	GA	TT	TT
**382**	GA	TT	CC	AA	CT	AA	AG	GA	CC	CC
**383**	GA	TT	CA	AA	CT	AC	GG	GG	CT	CT
**384**	GA	CT	CC	AA	CC	AC	AG	GG	CC	TT
**385**	GA	CT	AA	AA	CC	AA	AG	GG	CT	CT
**386**	GG	CT	CC	GG	CT	AA	AG	GG	CT	CT
**387**	GA	CC	CC	GG	CT	AA	AG	GG	CT	CT
**388**	GA	CT	CA	GA	CC	CC	AG	GA	CT	TT
**389**	GG	CT	CA	GA	CC	AA	AA	GG	CT	CT
**390**	GG	CT	CC	AA	CC	AC	GG	GG	CT	CT
